# Altering Visual Perception Abnormalities: A Marker for Body Image Concern

**DOI:** 10.1371/journal.pone.0151933

**Published:** 2016-03-22

**Authors:** Francesca L. Beilharz, Kelly J. Atkins, Anna J. F. Duncum, Matthew E. Mundy

**Affiliations:** School of Psychological Sciences and Monash Institute of Cognitive and Clinical Neurosciences, Monash University, Clayton, Victoria, Australia; University of Akron, UNITED STATES

## Abstract

The body image concern (BIC) continuum ranges from a healthy and positive body image, to clinical diagnoses of abnormal body image, like body dysmorphic disorder (BDD). BDD and non-clinical, yet high-BIC participants have demonstrated a local visual processing bias, characterised by reduced inversion effects. To examine whether this bias is a potential marker of BDD, the visual processing of individuals across the entire BIC continuum was examined. Dysmorphic Concern Questionnaire (DCQ; quantified BIC) scores were expected to correlate with higher discrimination accuracy and faster reaction times of inverted stimuli, indicating reduced inversion effects (occurring due to increased local visual processing). Additionally, an induced global or local processing bias via Navon stimulus presentation was expected to alter these associations. Seventy-four participants completed the DCQ and upright-inverted face and body stimulus discrimination task. Moderate positive associations were revealed between DCQ scores and accuracy rates for inverted face and body stimuli, indicating a graded local bias accompanying increases in BIC. This relationship supports a local processing bias as a marker for BDD, which has significant assessment implications. Furthermore, a moderate negative relationship was found between DCQ score and inverted face accuracy after inducing global processing, indicating the processing bias can temporarily be reversed in high BIC individuals. Navon stimuli were successfully able to alter the visual processing of individuals across the BIC continuum, which has important implications for treating BDD.

## Body Image

There are many perceptions of the self, however none as important as the continuously changing image of our own body. Body image is made up of the subjective cognitions, attitudes and emotions a person holds regarding their appearance [1]. When this perceived image does not meet their ideal an individual may experience body image concern (BIC; [2]), a phenomenon that is increasingly common in Western cultures [3]. This growing dissatisfaction is particularly concerning as BIC is implicated in a variety of mental health issues, from self-esteem to severe psychiatric conditions [4]. As such, BIC can be conceptualised as existing upon a continuum; ranging from a healthy and positive self-evaluation, across dissatisfaction, to a distorted and clinically significant body image conception [5]. Consequently, all individuals lie somewhere on the BIC continuum, depending upon their levels of distress.

At the most severe end of the BIC continuum is body dysmorphic disorder (BDD); a debilitating psychiatric condition characterised by clinically significant dysmorphic concern, or excessive distress regarding one’s appearance [6]. As defined by *The Diagnostic and Statistical Manual of Mental Disorders* [7], individuals living with BDD are preoccupied with their appearance, due to perceived physical flaws, which are slight or unobservable to others.

### Abnormal Visual Processing in Body Dysmorphic Disorder

Despite the debilitating consequences of BDD, the aetiology of this disorder remains relatively unknown [8]. Although many proposed factors may contribute to the development and maintenance of BDD [9–11], clinical observations alongside neurobiological and psychophysical research suggest that individuals with BDD tend to focus on the details of their appearance at the expense of their overall image [12]. This suggests that abnormalities in visual perception mechanisms, specifically global and local processing, may be involved in the onset and maintenance of BDD [13,14]

Global and local processing are the two mechanisms primarily involved in the identification and recognition of visual stimuli [15]. Local processing involves identifying a stimulus from the individual elements that comprise it, rather than its overall form, the latter of which refers to global processing [16,17]. In healthy individuals, most stimuli are initially processed globally, before reverting to a local technique if required by the stimulus [18]. Individuals with BDD however, have demonstrated a propensity to engage in predominantly local rather than global processing techniques regardless of the stimulus processing demands, suggesting there is an imbalance between these two systems [11–12,19–23].

The imbalance between global and local processing in BDD patients has been evidenced by patterns of brain activation using functional magnetic resonance imaging (fMRI). For example, previous research has demonstrated that when presented with face stimuli, BDD patients displayed hyperactivity in brain regions associated with local processing (e.g. the left hemisphere), and hypoactivity in regions known for global processing (e.g. primary and secondary visual areas) compared to healthy controls [21,22, 24]. These activation patterns suggest that individuals with BDD have a propensity to perceive visual stimuli using brain structures associated with local processing, irrespective of the type of information conveyed, whereas healthy individuals adapt their visual processing to specific stimulus demands [25–27].

A series of neuropsychological tests have provided further support for this local processing bias. For example, in a task focusing on facial appearance BDD participants displayed superior recognition compared to controls [11]. Similar findings of abnormal local and global information processing have emerged utilising the Rey-Osterrieth Complex Figure Test [19], an emotional face identity-matching task [27], variations of the Navon stimuli and embedded figures task [28], and eye tracking experiments [29,30]. Together, these results propose a dominance of local processing in BDD, which converges with evidence implicating disrupted face perception and atypical neural activation. However, as these studies were predominantly conducted upon individuals with a current BDD diagnosis, it remains unclear whether this local processing bias is a predisposing factor or a consequence of the disorder.

### Inversion Effects

Abnormal visual processing in individuals with BDD and high-BIC is further evidenced by research using inversion paradigms, in which stimuli are rotated 180° from their typical orientation. The face inversion effect is defined as the disproportional difficulty to recognise faces when they are upside down relative to upright [31]. Although most stimuli are harder to identify when upside down, faces are disproportionately impaired by inversion, demonstrated by less accurate and slower visual processing than their upright counterparts [16,32,33].

Faces are composed of a specific configuration of features, which are consistent across exemplars. Due to this familiarity, upright faces are processed globally or configurally [17], via the broad spatial relationships among the faces’ main components [34]. In a typical face, configural or global information refers to the relationship between two eyes located above a vertically centred nose and mouth [35]. Conversely, local or feature-based information refers to the individual elements of the stimulus, such as the unique size of the nose or eyes, which are processed in isolation [16,35,36]. Although both systems are needed to effectively process faces [37], global processing is especially important and occurs automatically [18,38]. It is global, rather than local information that is most sensitive to orientation and thus affected by inversion [35,37]. To recognise inverted faces individuals must switch from default global processing to local processing in order to identify the individual features, which subsequently causes delays and increased errors [17,26,31,32,34,39].

Although previous research has predominantly used face inversion paradigms, recent studies suggest that human body stimuli may be similarly affected. Human body stimuli are recognised via the spatial relations of specific body parts and how they connect to each other; typically a torso with two arms in the upper half and two legs in the lower half [33,34,40]. Reed et al. [33] presented strong evidence for a body posture inversion effect, as the inverted body stimuli were processed slower, and less accurately compared to the upright images of bodies. As such, it was inferred that human body stimuli are generally processed in a global manner, comparable to human faces [33,34,41].

Researchers have used these inversion effects to further demonstrate the local processing bias present in BDD patients, with results suggesting a decreased susceptibility to inversion effects compared to healthy controls. This phenomenon was illustrated by Feusner, Moller, et al. [12], who compared BDD patients to healthy matched controls in an upright-inverted facial discrimination task. A target facial stimulus was presented for either 500 or 5000 ms, followed by a second screen presenting the same face and a morphed version of the original. The task required participants to select the image matching the original face. Whilst all participants demonstrated a face inversion effect, as shown by an average increase in reaction time for inverted compared to upright faces, this effect was significantly reduced for BDD participants. As hypothesised, this result only occurred in the long stimulus presentation condition [12], which was due to the short duration stimuli affording only immediate and automatic global processing techniques to occur, whereas longer presentation times displayed the superior local processing of BDD patients [38]. This finding is supported by previous research using only short stimulus times [31]. Similarly, BDD patients had superior accuracy rates compared to healthy matched controls when asked to verbally identify inverted faces of famous celebrities [23]. These psychophysical studies provide additional evidence for a local processing bias, as those diagnosed with BDD have demonstrated both faster and superior recognition of inverted face stimuli, compared to healthy participants whose propensity for global processing impairs their response to inverted faces.

### Navon Stimuli

**Fig 1:**
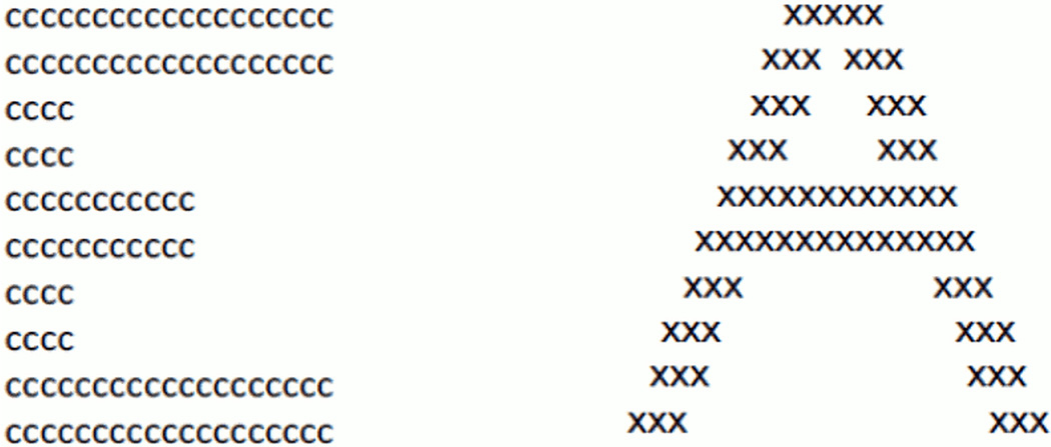
Navon stimuli. The image on the left consists of a global “E” made up of local “c” elements. The image on the right consists of a global “A” made up of local “x” elements.

A multitude of studies have demonstrated that presenting Navon stimuli [18] can alter visual processing [39,42–44]. These stimuli consist of a global figure, typically a large letter, made up of local elements, usually smaller incongruent letters (Fig 1;[36]). To induce processing of either global or local form, participants are presented with a series of Navon stimuli and are required to respond to either the large letter shape (global processing) or to the smaller letters within (local processing;[45,46]).

Navon-induced global processing has improved the subsequent recognition of faces viewed via videotape and in real world interactions [43,44], which has been replicated using both Navon letters and Navon inspired shapes [39,47]. These results indicate that inducing participants to process globally can subsequently improve face recognition which relies upon configural processing. Conversely, focusing on the local features of a Navon stimulus is believed to increase one’s propensity to process features of a face, rather than the configural whole [39]. This has been demonstrated using composite face paradigms, in which participants are required to correctly identify facial features. Detailed face recognition in the local Navon conditions was significantly faster than global conditions, indicating that induced local processing improves facial feature recognition [46]. Furthermore, a shift to global processing diminished performance on composite face tasks [48], suggesting that tasks requiring the processing of isolated features are impaired by inducing global processing. Despite the effect on subsequent global and local processing, no study to date has attempted to use Navon stimuli to alter the processing of individuals with high-BIC or clinically significant BDD.

### Local Bias as a Marker for BIC

As previous studies have focused on the local bias apparent in individuals with BDD, it remains to be seen whether this abnormality in visual processing can be considered an associated factor of this disorder [12]. As such, it is unclear whether this local processing bias precedes and thus contributes to the intense focus on details as reflected in BDD symptomology, or whether this maladaptive fixation on perceived defects later influences where vision is directed. Accordingly, Mundy and Sadusky [26] investigated whether inversion effects differed between non-clinical participants categorised as having high- or low-BIC. The Dysmorphic Concern Questionnaire (DCQ;[6]), a common BDD screening tool, was used to quantify BIC [49]. Extending from Feusner et al [12] by utilising both symptom specific and non-symptom related stimuli, both groups completed an upright-inverted stimulus (faces, bodies and scenes) discrimination task where participants discriminated between pairs of successive upright or inverted stimuli which were either the same image, or were morphed to be slightly different. This task revealed that compared to the low-BIC group, high-BIC participants were significantly faster at discriminating inverted faces and bodies, and had superior accuracy rates for discriminating inverted body and scene stimuli [26]. Although both groups demonstrated general face and body inversion effects, this was significantly reduced for the high-BIC individuals, reflecting visual processing deficits found in BDD participants [12,20]. It was concluded that as individuals presumed to be “at risk” of BDD displayed reduced inversion effects, this bias in local processing is a potential predisposition [26].

### The Present Study

Given the incomplete knowledge concerning the causes and subsequent treatment and prevention of BDD, there is a need to determine if a local processing bias is associated with varying levels of BIC in a non-clinical population. If this visual processing bias is indeed an associated factor of BDD-like symptoms, it is reasonable to suggest that as BIC increased along the continuum, a local processing bias would similarly increase in a graded manner. The present study examined how individuals across the entire BIC continuum differ in their visual perception, in order to determine if a local bias in visual processing could be a possible marker of BDD. Although the methodology of this study was primarily drawn from Mundy and Sadusky [26], the stimulus presentation times were increased to be similar to those of Feusner, Moller, et al. [12]. The Dysmorphic Concern Questionnaire was again used to robustly measure BIC. To examine the whole BIC continuum, participants with scores spanning the entire range of the DCQ completed the face and body stimulus upright-inverted discrimination task. In doing so, this study extended the findings of Mundy and Sadusky [26] from extreme dichotomous groups to a continuous sample, allowing for a greater generalisation across the range of naturally occurring BIC. Of additional interest was whether the expected local processing bias could be reduced using Navon stimuli. If so, the same finding may extend to individuals with a clinical diagnosis of BDD, which has potential treatment implications [23].

Based on previous findings that individuals with BDD and high-BIC displayed a local bias in visual perception, it was hypothesised that BIC would be associated with a local processing bias, as shown by reduced inversion effects. Specifically, this bias would be demonstrated by a positive relationship between DCQ scores and accuracy rates (%) on the face as well as body stimulus discrimination tasks, and a negative relationship between DCQ scores and reaction time (milliseconds) on the same tasks. These relationships were presumed to show that individuals with high-BIC would have faster and superior processing on inverted face and body tasks, and therefore process in a more local manner.

Exposure to Navon stimuli however, was expected to manipulate this effect. It was hypothesised that after inducing global processing using Navon stimuli, a negative correlation would be found between DCQ score and accuracy rates on both the inverted face and body stimuli tasks, as participants at the higher end of the BIC continuum would be more affected by an induced bias to global processing (due to the assumption that global processing is less used in everyday life in these individuals, compared with lower-BIC). It was also expected that inducing local processing would have the opposite effect; specifically that a positive correlation would exist between DCQ score and accuracy rates on both the inverted face stimuli and body stimuli tasks, as those with lower BIC would improve at discriminating these stimuli when given the same processing advantage already present in those at the higher end of the BIC continuum. The overall aims of this research to determine if a local processing bias is a linked factor of BDD, and whether this bias could be altered using Navon stimuli were met.

## Method

### Participants

Participants were recruited from Monash University, Clayton Campus Australia. Volunteers completed an online version of the DCQ ([6]; see materials below). A total of 413 individuals participated in the online questionnaire, and of these 128 were invited via phone or email to participate in the behavioural portion of the study (see selection process as outlined below). From these invitations 74 individuals consented, giving a final sample comprising 64 females and 10 males (*M* age = 22.97, *SD* age = 6.66 years). Two participants reported a previous diagnosis of both BDD and an eating disorder, while five other participants had been diagnosed with an eating disorder alone. This research was approved by the Monash Human Research Ethics Committee, and all participants provided informed written consent.

#### Selection process

Individuals were selected to represent the entire BIC continuum, therefore including scores across the entire range of the DCQ. Due to time and financial constraints, a sample was randomly selected representing this continuum with approximately even numbers of participants scoring in each quartile of the DCQ range. The behavioural study comprised two phases, each involving a separate discrimination task. All participants completed both phases and received $20 as compensation for their time.

### Materials

The online version of the DCQ was presented using Qualtrics. Participants responded to a series of demographic and screening questions to assess eligibility, and provided their contact information before the DCQ items were presented.

Participants also completed the Body Image Concern Inventory [50], the Eating Disorder Inventory [51] and the SCOFF Questionnaire [52], the results of which are not presented here.

#### Dysmorphic Concern Questionnaire

The DCQ measures levels of dysmorphic concern and is a screening tool for BDD [48]. This self-report questionnaire has 7 items rated on a 4-point Likert-type scale (from *0 = “Not at all”* to *3 = “Much more than most people”)*. Possible scores range from 0–21, with a cut-off score of 9, and higher scores indicating greater BIC. The DCQ items ask individuals about how they perceive their physical appearance and their associated behaviours, relative to others. This valid and sensitive measure has Cronbach’s alpha scores ranging from .80-.88, demonstrating good internal consistency [6,53].

#### Stimuli

The face stimuli were created from colour photographs sourced from the Psychological Collection of Images at Stirling database (available at pics.stir.ac.uk) and the Centre for Vital Longevity Face Database ([54] available at agingmind.utdallas.edu/facedb). These front-on faces were emotionally neutral, and comprised both male and female faces from a range of ethnicities. Original images of the same gender with similar facial features were paired together to assist the morphing process. Using Morpheus Photo Morpher [55], each pair of original faces were morphed to create a combined face image, which was then paired with the original image it most closely resembled to create a unique pair. From this unique pair, there were four possible outcomes, of which the “same” matching pairs were made of the original stimulus and its exact copy, or the morphed stimulus and its exact copy; while the “different” non-matching pairs comprised the original stimulus and the morphed stimulus, or vice versa. There were 16 unique face pairs, giving a total of 64 possible outcomes. The morphing of stimuli was designed to increase discrimination difficulty, with the difficulty level based on stimuli utilised by Mundy and Sadusky [26]. The 16 unique face pairs were cropped and resized so that all stimuli were 325*500 pixels, and any extraneous details and morphing artefacts were eliminated.

**Fig 2:**
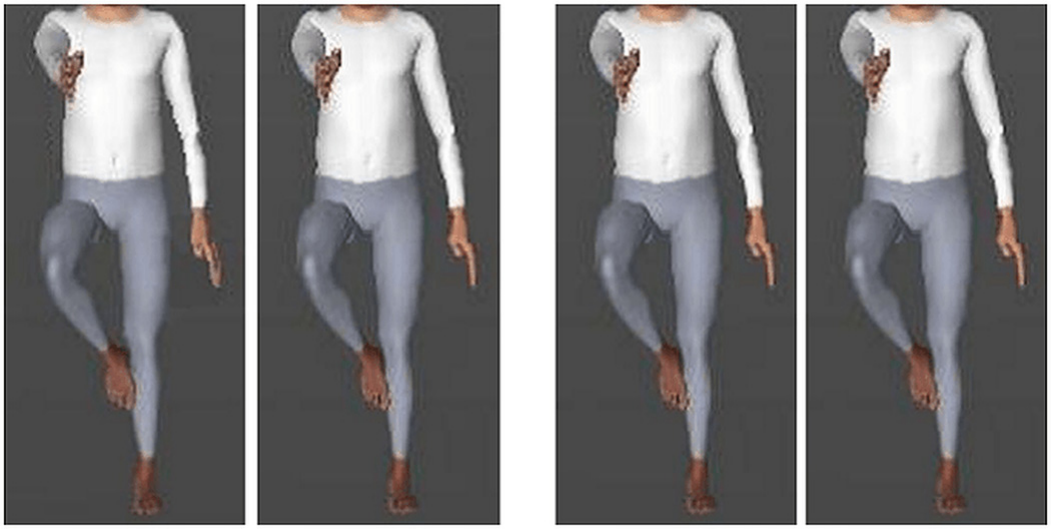
Example of upright non-matching (left) and matching (right) body pairs used in the discrimination task. Please see [57] for an example of the face stimuli.

The clothed body stimuli were computer generated colour images of male and female virtual reality human bodies in a three-dimensional environment, created using HumanCAD [56]. Images roughly similar in body posture were morphed using Morpheus Photo Morpher [55], using the same process as for faces. The 16 unique body pairs were considered comparable in difficulty to stimuli used by Mundy and Sadusky [26]. As with the face stimuli, the 16 unique body pairs had 64 possible outcomes. The body stimuli were resized to 200*500 pixels, and the heads were cropped out to prevent possible face effects. An example of non-matching and matching face and body stimulus pairs may be seen in Fig 2. Eight unique face pairs and eight unique body pairs were used in phase one, whilst the remaining 8 unique face and body pairs were used in phase two to prevent practice effects.

The behavioural task was run on an IBM compatible PC, using Presentation [58] to record participants’ average accuracy rates (%) and response times (ms).

Phase two included 26 Navon letters (e.g. an H composed of Qs), with each large letter figure made of smaller incongruent letters in Arial font type, size 18. Navon letters were cropped and resized to 200x200 pixels, with black text against a white background. All 26 letters in the English alphabet were used twice, once as a global letter figure and once as a local feature.

### Procedure

Participants completed the online questionnaire, with their subsequent DCQ scores used as selection criteria for the behavioural experiment. Participants were seated approximately 70cm away from an eye-level 21 inch PC computer monitor and instructed that one image would appear on the screen, followed quickly by another image. The task required participants decide as quickly and accurately as possible whether these images were different or the same.

For each trial, the first stimulus of the pair was presented for 1500 ms, followed by a blank screen for 300 ms, after which the second stimulus appeared for 1500 ms. The response time began 300 ms into the presentation of the second stimulus, and responses before this period were discarded as anticipation errors. Once the second stimulus disappeared it was replaced by an intertrial blank screen for a further 1800 ms, limiting response times to 3000 ms for each stimulus pair. If no response was made during this period, the next trial began automatically, and the non-response trial was discarded. The time between the end of one trial and the beginning of another varied between 500 to 3000 ms.

In phase one of the experiment (*n* = 74), each participant was shown eight unique pairs of faces, and eight unique pairs of bodies, which were presented in separate blocks of 48 trials each, giving a total of 96 trials per participant. The block order of faces and bodies was randomly counterbalanced across participants. Within each block, half of the stimulus pairs were presented inverted, while the other half were upright. The two stimuli composing a pair were always matching in orientation, and the selection of upright and inverted stimuli from the eight unique pairs was randomised across participants. Each unique pair was shown a total of six times, with half of the presentations requiring “same” as the correct response for a matching pair, and the other half requiring “different” as the correct response for a non-matching pair. Additionally, the order of trials within each block was randomised, giving a total testing period of approximately 20 minutes for each participant. Responses in phase 1 acted as a control for the Navon manipulation in phase 2.

The second phase of the experiment followed the same paradigm as the first, however the face and body stimulus presentations were separated into three blocks. Between each of these blocks, a series of Navon stimuli were presented. Each of the six Navon stimuli blocks contained 48 letters, for a total of 228 presentations. Each letter was presented for 1 s, with a 1 s inter-stimulus interval blank screen dividing each presentation. The repeated presentation of Navon stimuli blocks ensured that the effect of the Navon stimuli did not diminish throughout subsequent face and body stimuli trials [36,59]. In phase 2, participants were divided into two conditions, and were instructed to focus on either local (*n* = 38) or global (*n* = 36) Navon stimuli. The second phase of the experiment took approximately 20 minutes.

## Results

In both phases of the study, the number of correctly discriminated trials (calculated as a percentage) and discrimination reaction time (RT; calculated in milliseconds) were recorded for inverted and upright face trials. Within the RT data, only trials in which the discrimination response was correct were analysed. All statistical analyses were run through SPSS Statistics version 20 (IBM, Australia) for Windows, with an alpha level of .05, two-tailed. Prior to all statistical analyses, assumptions were met, and the presence of univariate outliers were assessed and adequately dealt with ensuring all z-scores were within ±3.29.

### Phase One

The mean DCQ score, accuracy rates and reaction times of this sample may be seen in Table 1.

**Table 1:** Means, standard deviations and 95% confidence intervals for all variables.

	*Mean (SD)*	*95% CI*
DCQ Score	8.32 (6.09)	[6.91, 9.73]
Upright Face Accuracy (%)	78.45 (7.86)	[76.64, 80.28]
Upright Face Reaction Time (ms)	734.62 (145.82)	[700.84, 768.41]
Inverted Face Accuracy	67.12 (9.02)	[65.03, 69.21
Inverted Face Reaction Time (ms)	731.23 (138.24)	[699.20, 763.26]
Upright Body Accuracy (%)	78.15 (8.27)	[76.23, 80.06]
Upright Body Reaction Time (ms)	762.58 (149.56)	[727.93, 797.23]
Inverted Body Accuracy (%)	68.27 (5.62)	[66.97, 69.57]
Inverted Body Reaction Time (ms)	723.11 (145.75)	[689.34, 756.88]

**Fig 3:**
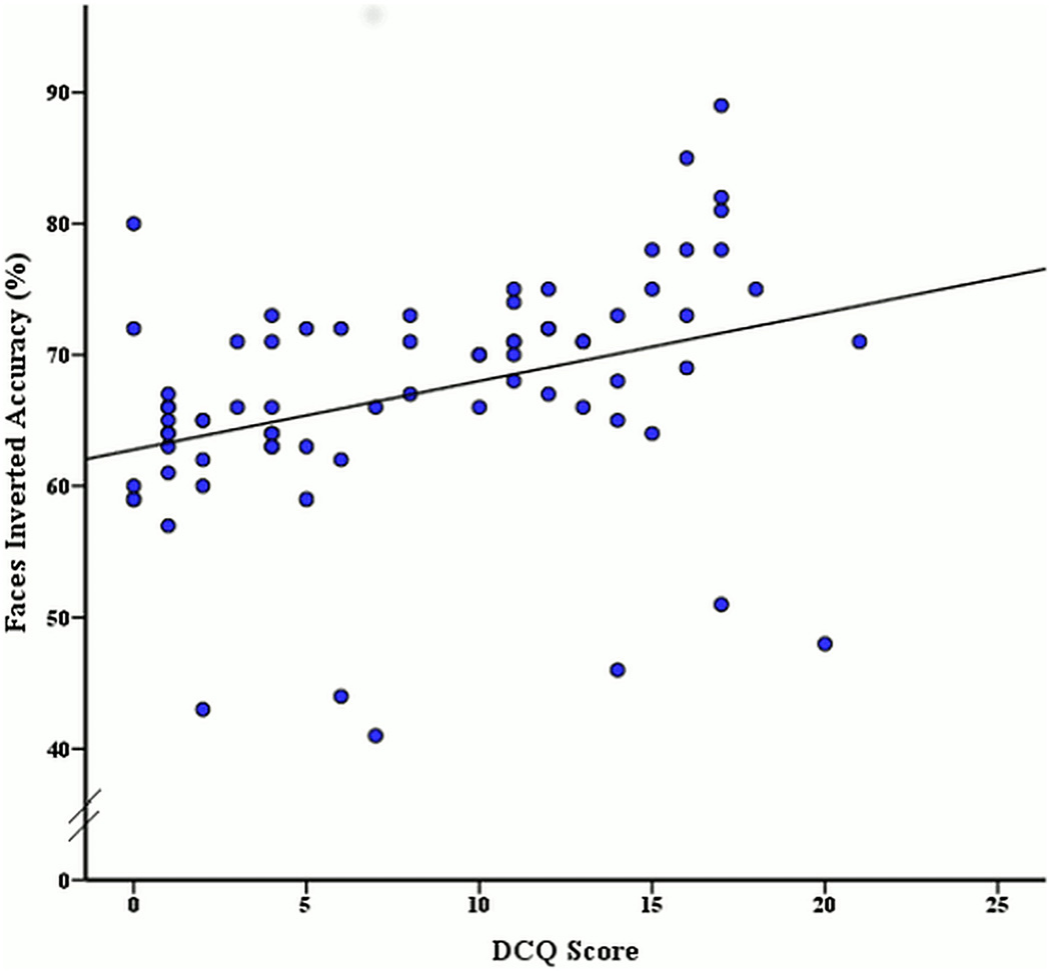
Relationship between DCQ score and mean accuracy rates (%) of inverted face stimuli discrimination.

**Fig 4:**
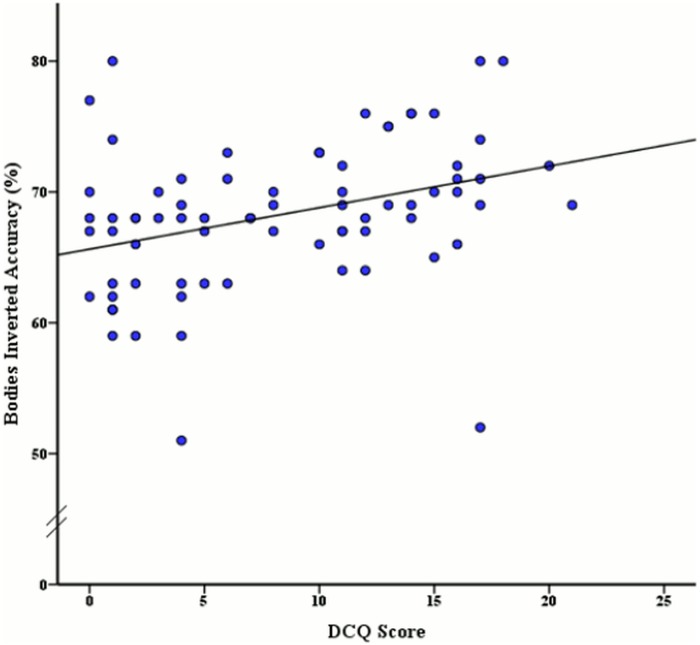
Relationship between DCQ Score and mean accuracy rates (%) of inverted body stimuli discrimination.

**Fig 5:**
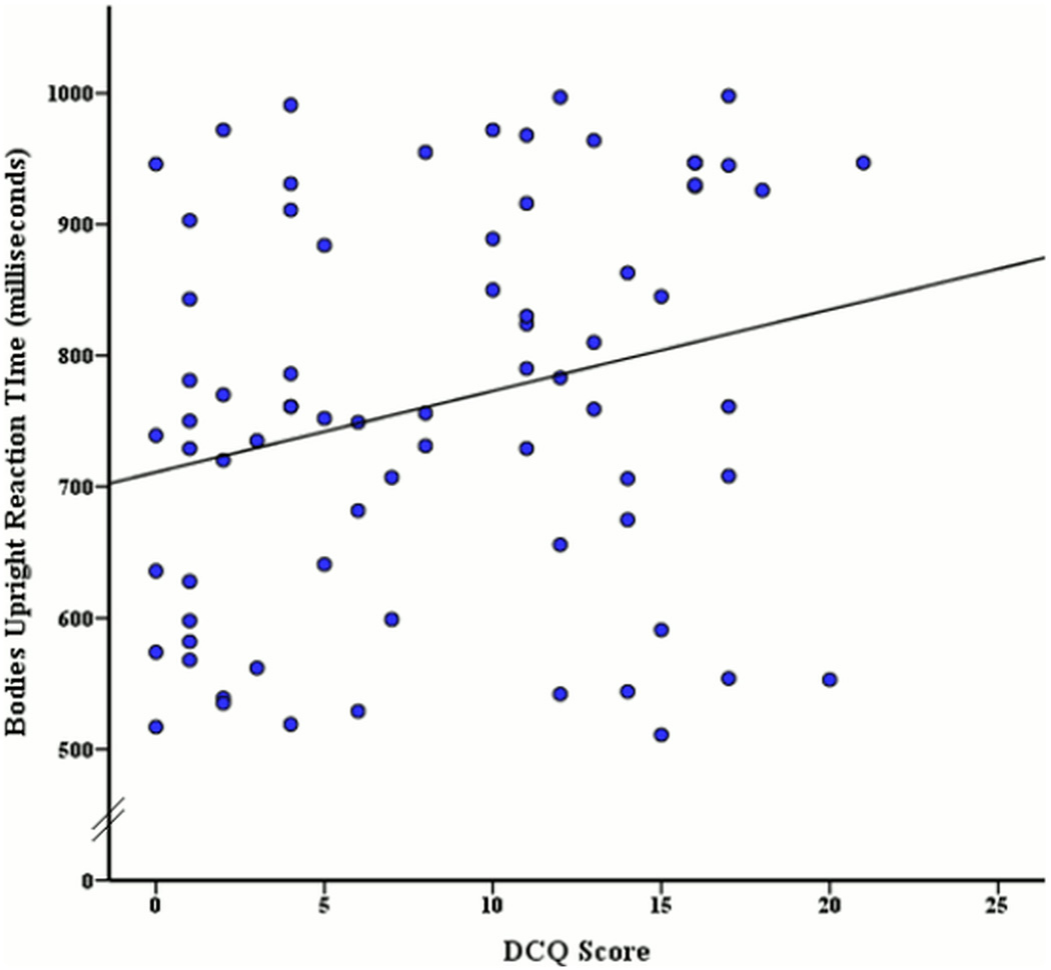
Relationship between DCQ score and mean reaction times (ms) of upright body stimuli discrimination.

To determine if accuracy rates and reaction times for face and body stimuli were significantly related to varying levels of BIC, a Pearson correlation coefficient analysis was used. The Pearson correlation revealed a significant moderate positive relationship between the DCQ score and inverted face accuracy, *r* (74) = .35, *p* < .001, as illustrated in Fig 3. A significant moderate positive relationship was also found between DCQ score and inverted body accuracy *r* (74) = .34, *p* = .003 (see Fig 4). Additionally a moderate positive relationship between BIC score and upright body reaction time was revealed, *r* (74) = .25, *p* = .03, as seen in Fig 5. No other correlations were significant, although the remaining correlations with DCQ score may be seen in Table 2.

**Table 2:** Pearson Correlation Coefficients between DCQ Scores and Behavioural Performance for each of the test conditions.

	Faces		Bodies	
	Upright	Inverted	Upright	Inverted
Accuracy (%)	- .14	.35**	.06	.34**
Reaction Time (ms)	- .18	- .03	.25*	.06

** = *p* < .01,

* = *p* < .05, two-tailed

### Phase Two

#### Global Processing

**Fig 6:**
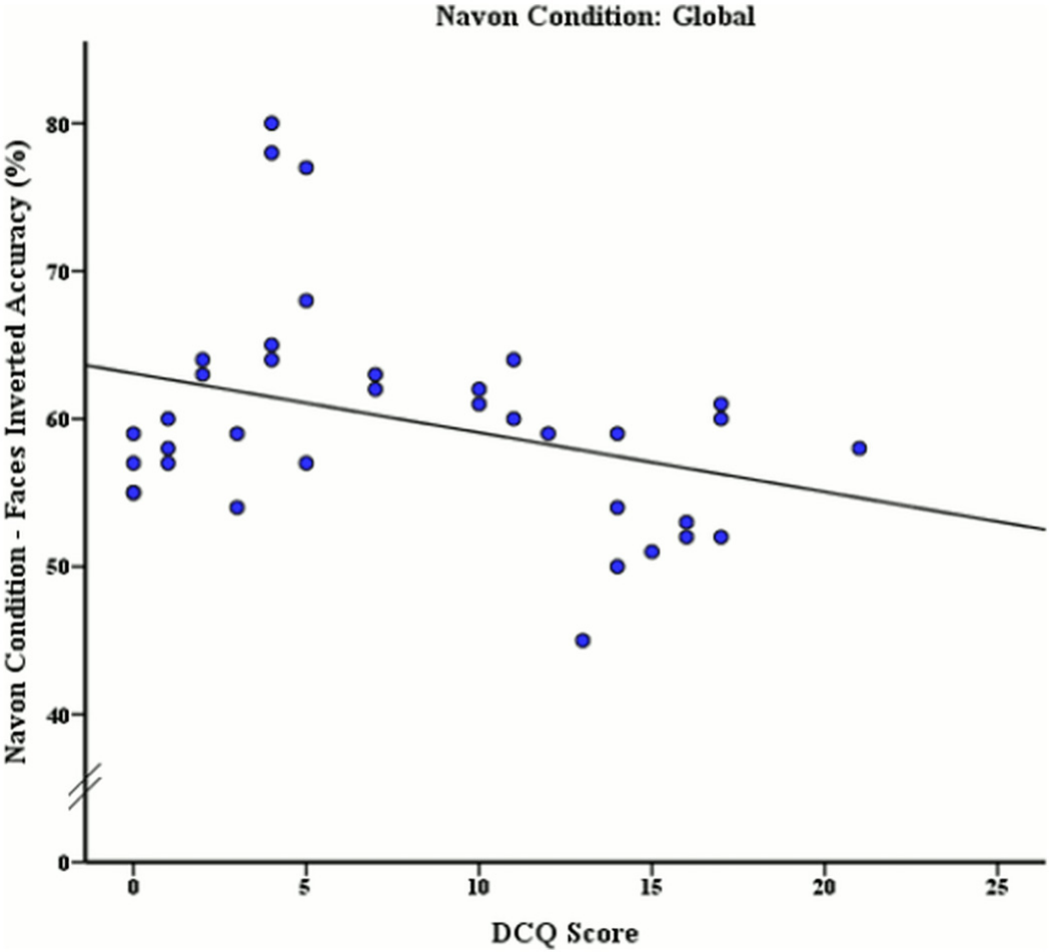
Relationship between DCQ score and mean accuracy rates (%) of inverted face stimuli discrimination after global processing was induced.

The mean DCQ score, accuracy rates and reaction times of this sample by global Navon condition may be seen in Table 3. To determine if accuracy rates and reaction times for face and body stimuli were significantly related to varying levels of BIC after global processing had been induced using Navon stimuli, a Pearson correlation coefficient analysis was used. The Pearson correlation revealed a significant moderate negative relationship between the DCQ score and inverted face accuracy, *r* (36) = -.34, *p* = .042, as illustrated in Fig 6. To examine whether the correlations between phase 1 and phase 2 of the experiment were significantly different, a z-test was used after converting correlation coefficients to z scores. This z-test revealed that accuracy scores for inverted face stimuli were significantly decreased without Navon manipulation compared to global Navon manipulation, *z* = 3.42, *p* < .001.

**Table 3:** Means, standard deviations and 95% confidence intervals for all variables in the global Navon condition.

	*Mean (SD)*	*95% CI*
DCQ Score	7.94 (6.28)	[5.82, 10.07]
Upright Face Accuracy (%)	79.39 (9.14)	[76.29, 82.48]
Upright Face Reaction Time (ms)	772.33 (147.44)	[722.45, 822.22]
Inverted Face Accuracy (%)	59.89 (7.41)	[57.38, 62.40]
Inverted Face Reaction Time (ms)	745.75 (130.87)	[701.47, 790.03]
Upright Body Accuracy (%)	81.50 (5.94)	[79.49, 83.51]
Upright Body Reaction Time (ms)	713.08 (151.90)	[661.69, 764.48]
Inverted Body Accuracy (%)	58.86 (5.04)	[57.15, 60.57]
Inverted Body Reaction Time (ms)	758.67 (136.88)	[712.35, 804.98]

#### Local Processing

**Fig 7:**
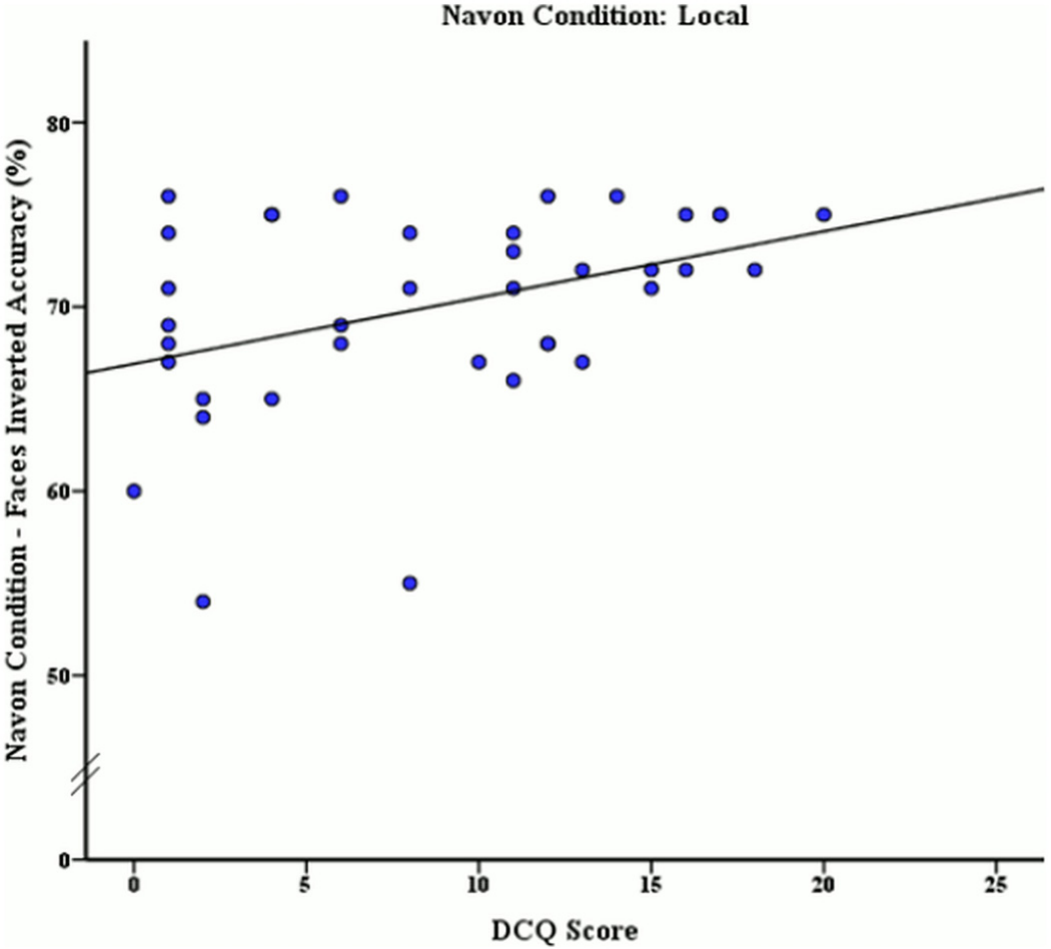
Relationship between DCQ score and mean accuracy rates (%) of inverted face stimuli discrimination after local processing was induced.

The mean DCQ score, accuracy rates and reaction times of this sample by local Navon condition are presented in Table 4. A Pearson correlation coefficient analysis was also used to examine the relationships between BIC and accuracy rates and reaction times for face and body stimuli after local processing had been induced using Navon stimuli. This correlation analysis revealed a moderate positive significant association between DCQ score and inverted face accuracy *r* (38) = .39, *p* = .015, as seen in Fig 7. To determine whether the correlations between phase 1 and phase 2 of the experiment were significantly different, a z-test was used after converting correlations coefficients to z scores. This z-test revealed that after local Navon manipulation, accuracy performance for inverted face stimuli was not significantly different to performance prior to Navon manipulation, *z* = -.22, *p* = .82.

**Table 4:** Means, standard deviations and 95% confidence intervals for all variables in the local Navon condition.

	*Mean (SD)*	*95% CI*
DCQ Score	8.68 (5.96)	[6.73, 10.64]
Upright Face Accuracy (%)	72.92 (9.21)	[69.89, 75.95]
Upright Face Reaction Time (ms)	771.39 (137.57)	[726.18, 816.61]
Inverted Face Accuracy (%)	70.03 (5.45)	[68.24, 71.82]
Inverted Face Reaction Time (ms)	726.42 (136.19)	[681.66, 771.19]
Upright Body Accuracy (%)	74.89 (5.69)	[73.02, 76.76]
Upright Body Reaction Time (ms)	722.92 (149.87)	[673.66, 772.18]
Inverted Body Accuracy (%)	72.26 (7.61)	[69.76, 74.77]
Inverted Body Reaction Time (ms)	732.39 (140.13)	[686.34, 778.45]

**Fig 8:**
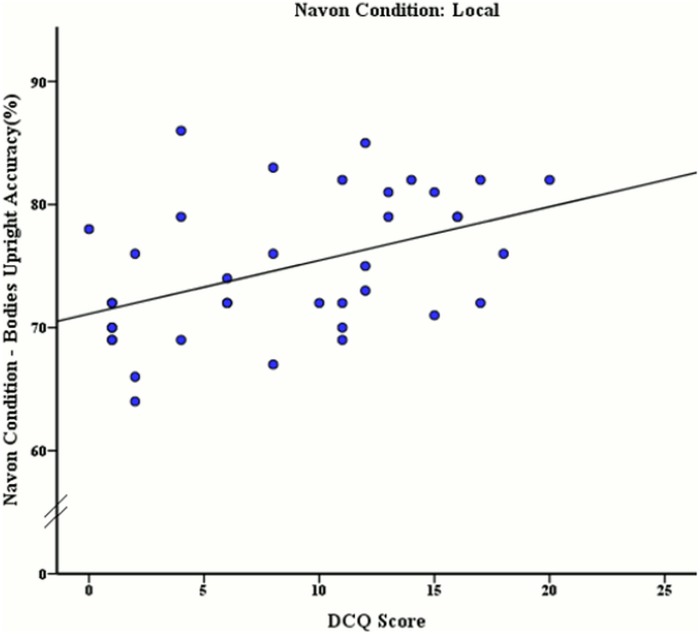
Relationship between DCQ score and mean accuracy rates (%) of upright body stimuli discrimination after local processing was induced.

A moderate positive significant relationship was also discovered between DCQ score and accuracy rates for upright body stimuli *r* (38) = .46, *p* = .004 (see Fig 8). All other correlations with DCQ score may be seen in Table 5.

**Table 5:** Pearson Correlation Coefficients between DCQ Scores and Behavioural Performance for each of the test conditions in both Local and Global Navon groups.

	Faces		Bodies	
	Upright	Inverted	Upright	Inverted
Global				
Accuracy (%)	.02	-.34*	.29	- .08
Reaction Time (ms)	.13	.06	-.14	.25
Local				
Accuracy (%)	.18	.39*	.46**	.26
Reaction Time (ms)	-03	- .09	.09	.32

** = *p* < .01,

* = *p* < .05, two-tailed

It should be noted that an identical set of Pearson correlation coeffecient analyses were conducted using participants’ scores on the Body Image Concern Inventory (BICI; Littleton et al., 2005), and a composite measure of the DCQ and BICI (DCQ score x 4.52 + BICI), in place of the DCQ measure used above. However, all scores using these measures were significantly related with strong positive relationships between DCQ scores and BICI scores *r* (74) = .91, *p* < .00, DCQ scores and BIC Composite scores *r* (74) = .98, *p* < .00, and BIC scores and BIC Composite scores *r* (74) = .97, *p* < .00. The same significant results with the discrimination task were also found using the BICI and composite scores, thus only the DCQ results were reported given this measure has been previously used within this research area. However, these findings provide additional support for the external validation of these measures.

## Discussion and Conclusion

The present study investigated whether a local bias in visual processing was associated with the development of body dysmorphic disorder, and whether this potential bias could be altered using Navon stimuli. The hypothesis that increases in BIC, as defined by DCQ score, would be linked to reduced face and body inversion effects, manifest by superior accuracy rates and faster reaction times for inverted stimuli, was supported by participant accuracy rates. Furthermore, results also partially supported the hypothesis that inducing global or local processing would lead to decreased or increased accuracy rates for inverted face and body stimuli respectively, across the BIC continuum.

The results of phase 1 demonstrated positive significant moderate relationships between DCQ scores and accuracy rates for the inverted face and body stimuli. This indicates that a graded local bias accompanied increases in BIC, thus supporting the suggestion that this visual processing bias may be a predisposing feature, occurring before a potential BDD diagnosis.

Additionally, an unexpected association was revealed between DCQ score and reaction time for upright body stimuli in the discrimination task, indicating that as individuals increased in BIC, so did the amount of time spent processing the upright body images. This unexpected relationship has not previously been demonstrated, as BDD participants and healthy controls (including those with high- and low-BIC) have formerly shown relatively similar response times to this stimulus type [12,26]. It is possible that this longer looking duration for upright bodies was related to the high-BIC and BDD tendency of comparing one’s appearance to those of other people, as these individuals often spend a debilitating amount of time examining their own appearance in this typical orientation [60]. However, this association should be interpreted cautiously.

The relationship between the BIC continuum and a local processing bias is consistent with previous research, as this visual perception abnormality has been suggested as a possible marker of BDD [26]. This association supports previous findings, as high-BIC participants have previously demonstrated reduced inversion effects [26]. Mundy and Sadusky [26] also noted differences between low- and high-BIC groups in the speed of inverted face and body processing, associations of which were not found here. These inconsistent findings may be explained by a possible trade-off between participants’ accuracy rates and reaction times. It is presently unclear what contributed to this trade off, with differing stimulus presentation times or phrasing of task instructions potentially contributing to more variable response times. Nonetheless, it appears that in the current study participants valued accuracy over reaction time.

While the current research could not explicitly determine whether this local bias contributes to the development of BDD, evidence primarily supports the presence of this visual abnormality in BDD patients. Feusner, Moller, et al. [12] noted faster reaction times, while Jefferies et al. [23] found superior recognition for inverted face stimuli compared to healthy control participants. Combined with this psychophysical evidence of reduced inversion effects, brain imaging [21,22,61] and neuropsychological test results [11,19,27,28] provide additional support for a dominance of local processing and superior visual performance in BDD. There is some contention that the excessive preoccupation typical of BDD is not caused by a visual processing abnormality [32], but as only two participants in the current sample had a self-reported BDD diagnosis this could not be explicitly addressed. Nevertheless, it is expected that individuals with BDD have atypical visual perception, with current evidence indicating a local processing bias could be one of many associated factors.

This study also investigated whether the visual processing of individuals across the BIC range could be altered using Navon stimuli. It was hypothesised that using Navon stimuli to induce global processing would result in a negative relationship between DCQ scores and accuracy rates, and a positive association between DCQ scores and reaction times, for inverted face and body stimuli. This hypothesis was partially supported, as there was a significant moderate negative relationship between DCQ score and accuracy rates for the inverted face stimuli after participants were induced to process globally. This correlation was significantly different to that in phase 1, suggesting that globally biasing participants was successful in altering participants’ visual processing. This indicates that global biasing significantly worsened inverted face perception as BIC levels increased (processing an inverted face globally prevents attention to feature details that are critical to inverted face perception). Therefore, individuals at the higher end of the BIC continuum appeared to be more affected by the induced global processing, resulting in increased face inversion effects. This greater susceptibility to global biasing in higher BIC levels may be due to the fact that these individuals are less accustomed to this form of processing. As expected, the opposite effect was also found: after local biasing was induced, a moderate positive relationship was found between DCQ score and accuracy rates for inverted face stimuli. Therefore, local biasing improved inverted face recognition as BIC increased, demonstrating decreased inversion effects at the higher end of the BIC continuum. However this correlation was not significantly different to that in phase 1, and so we cannot say that local biasing using Navon stimuli successfully altered participants’ responses. For this relationship, there appeared to be a ceiling effect, as participants did not score above 80% in the inverted face stimulus discrimination task, yet there was greater variability in the accuracy rates of those with low-BIC compared to those with high-BIC. This is presumably due to the fact that high BIC participants already had a local bias in place, and inducing further local processing was unable to improve their abilities due to their already high results (as seen in phase 1).

Unexpectedly, a significant relationship was also found between DCQ score and accuracy rates for upright body stimuli after local processing had been induced, indicating that local biasing improved recognition of upright bodies as BIC increased. It is possible the unexpected relationships concerning upright body stimuli may have been due to some feature of the body stimuli used in this study that favoured local discrimination as opposed to global. While there was still an inversion effect, as participants performed worse when body stimuli were inverted compared to upright, it is possible that these stimuli were not primarily processed globally by healthy individuals as would typically be the case. This abnormality in body stimuli may also have affected the relationships with inverted body stimuli after inducing processing styles, as significant results were only found for the inverted face stimuli.

While no studies to date have altered the visual processing of those with varying levels of BIC, the current results are consistent with a large body of research indicating that Navon induced processing temporarily influences visual perception [39,42–44,46], and that experimentally inducing global processing impairs the perception of stimuli requiring a local technique [47].

### Limitations

There are some limitations which may have influenced the current findings. Firstly, different stimuli were used compared to previous studies, which may have contributed to some variation in these accuracy and reaction time results. Although the face and body pairs were designed to be equivalent in difficulty level, and were based on those of Mundy and Sadusky [26], this was not empirically assessed. Additionally, while participants were excluded who did not have self-reported normal vision, visual acuity was not tested in this sample, which may also have influenced some discrepancies between participant results.

Secondly, due to the nature of Navon induced processing, the first and second phases of the experiment could not be counterbalanced, which may have resulted in practice effects, limiting the impact of Navon manipulation. To overcome this limitation, future research should endeavour to increase the interval between discrimination tasks, allowing for the effect of Navon stimuli to diminish and thus enabling counterbalancing.

Furthermore, this study may have been limited by sampling methods, as eight participants self-reported an eating disorder diagnosis and less is known regarding visual processing in these disorders. Additionally, although the DCQ specifically measures dysmorphic concern (a primary symptom of BDD), these items may be interpreted in terms of weight and body size. However, of these eight participants, two reported concurrent BDD diagnoses, the inclusion of which represents a strength of the current study as we utilised a truly representative continuum of BIC, ranging from healthy to clinically diagnosable concerns. The current sample may also have been influenced by mood or symptoms of depression, both of which have been linked with changes in local and global processing [62,63], and also correlate with DCQ scores [6]. As the mood of participants was not measured, this would be an important inclusion for future research to reduce any impact this has on visual processing associated with levels of body image concern.

Finally, due to the correlational design, this study was unable to determine causation. Therefore, while abnormal visual processing appears to be linked to BIC in a graded manner, it remains unclear whether increasing BIC influences visual processing, or whether abnormalities in perception consequently affect how individuals perceive their appearance.

### Broader Implications

Despite these limitations, it appears that there are associated patterns of abnormalities in visual perception across the BIC continuum. The superior discrimination of inverted face and body stimuli accompanying increased levels of BIC, indicates a fixation on specific, local features of one’s physical appearance. Unquestionably, an array of factors are implicated in the development and maintenance of BIC-related conditions such as BDD [9–11], and further research is necessary to determine how much of an impact this marker may have, and how this interacts with other factors to influence the onset of BDD.

Nevertheless, findings of this kind may be key to the development of innovative early assessment and intervention strategies. For example, if a client presents with BIC, perhaps a psychophysical test could be utilised to infer their level of appearance-related concern. A face inversion task could potentially assess the extent to which individuals rely on global or local processing, which may then reflect their degree of distorted perception. This task would be of particular use for individuals with low insight into their mental health issues, as is frequently the case in BDD and other BIC-related disorders [64]. There is also potential for the implementation of imaging techniques to determine if a local processing bias is detectable at a neural level across varying BIC, although further neuroimaging research is necessary to determine whether this is present in pre-clinical BIC. Evaluating BIC in this psychophysical or neural manner would provide an objective measure at this earlier stage of assessment, and may assist in preventing the later onset of associated psychiatric conditions.

The findings of the current study could additionally influence treatment options, which may drastically lower the occurrence and severity of BIC and BDD. As graded levels of this local bias are associated with increasing BIC, it seems possible that altering one’s visual perception may affect how they perceive their physical appearance. The current findings indicated that using Navon stimuli to induce global processing lead to inferior inverted face perception as individuals increased in BIC, demonstrating a greater face inversion effect (and thus an increased reliance on global processing). This suggests that a move away from a pre-existing maladaptive local bias, towards a healthier and more normal global processing precedence may be possible after patients are induced to process globally via the use of Navon stimuli.

However, research is needed to establish whether the same effect is present in a clinical BDD sample, who have repeatedly demonstrated a reduced susceptibility to the face inversion effect [12,23]. Increasing the face inversion effect in this way may translate to a reduction in the local processing techniques typically utilised by this population, and thereby temporarily reduce the preoccupation with physical features. This maladaptive tendency to fixate on the individual details of their appearance is exacerbated by the failure to acknowledge these features within the context of their overall appearance [13,65]. Reductions in the visual processing of facial and bodily features may therefore be an important addition to current treatment programs to assist individuals in developing more accurate and adaptive beliefs regarding their appearance. This perceptual intervention may be implemented within perceptual retraining, which is a current component of cognitive-behavioural therapy for BDD [63], and may additionally reduce the distress and anxiety associated with the appearance dissatisfaction [30,66]. However future studies should also examine the duration and strength of any improvements in visual perception caused by global biasing in a clinical population.

### Concluding Remarks

The current research indicated that a local bias in visual processing is linked to BDD, which was supported by the moderate positive associations between BIC and superior accuracy rates for inverted face and body stimuli. Examining participants across the entire BIC continuum allowed greater ecological validity, as these findings can be extended to the wider population, of whom vary greatly in their levels of concern. Furthermore, this continuum analysis enabled the true dimensionality of BIC to be assessed, which has not previously been conducted in relation to visual processing.

These findings contribute to the growing understanding of BDD and associated abnormalities in visual perception, and provide growing support for this local processing bias as a marker. This general visual perception bias associated with varying levels across the BIC continuum has important implications regarding future prevention and treatment strategies. A greater understanding of contributing factors may assist in preventing the progression from high BIC to clinically concerning cases of BDD, which is imperative given the debilitating functional, emotional and psychological outcomes associated with this disorder.

Additionally, experimentally biasing participants to process globally or locally appeared to worsen or improve inverted face processing, respectively, as individuals increased in BIC. Given the subsequent increase in inversion effects displayed by those in the global Navon condition, the use of Navon stimuli with clinical BDD samples should be a focus of future research. As a preoccupation with facial and body features is a primary symptom of BDD, often leading to severe distress and debilitating appearance related anxiety, reducing this fixation has important treatment implications and clinical outcomes.

In order to more clearly establish that a local bias is implicated in the aetiology of BDD, a future longitudinal study could examine whether any increases or decreases in BIC level are accompanied by associated changes in visual perception, for instance, assessing whether the worsening of BIC symptoms to clinically diagnosable BDD is accompanied by a stronger local processing bias. Further research is also required into the genetic and attentional processes involved in BDD, which may clarify whether this local processing bias is a learned behavioural process or an inherited neurological flaw; an understanding of which may assist in the prevention and treatment of potentially harming body image concern.
